# Linking What We Eat to Our Mood: A Review of Diet, Dietary Antioxidants, and Depression

**DOI:** 10.3390/antiox8090376

**Published:** 2019-09-05

**Authors:** Qingyi Huang, Huan Liu, Katsuhiko Suzuki, Sihui Ma, Chunhong Liu

**Affiliations:** 1 College of Food Science, South China Agricultural University, Guangzhou 510642, China (Q.H.) (H.L.); 2 The Key Laboratory of Food Quality and Safety of Guangdong Province, Guangzhou 510642, China; 3 Graduate School of Sport Sciences, Waseda University, Tokorozawa 359-1192, Japan; 4 Faculty of Sport Sciences, Waseda University, Tokorozawa 359-1192, Japan

**Keywords:** depression, food, dietary pattern, nutrition, oxidative stress, inflammation, cytokine

## Abstract

Studies have shown that diet and nutrition play significant roles in the prevention of depression and its clinical treatment. The present review aims to provide a clear understanding of the associations between diet patterns, specific foods, nutrients such as antioxidants, and depression. As a result, balanced dietary patterns such as the Mediterranean diet and certain foods such as fish, fresh vegetables, and fruits have been associated with a lower risk of depression or depressive symptoms, while high-fat Western diets and sugar-sweetened beverages have been associated with higher risk of depression or depressive symptoms. Dietary antioxidants such as green tea polyphenols or isoflavonoid intake have been negatively associated with depression or depressive symptoms. It is concluded that diet patterns, specific foods, and antioxidants play important roles in the prevention and clinical treatment of depression.

## 1. Introduction

The World Health Report published by the World Health Organization (WHO) in 2017 identified that the global incidence of depression was over 18% between 2005 and 2015 [1]. Depression has now become the fourth major disease in the world [2], and is predicted to become the world’s second major contributor to the global burden of disease, only less than ischemic heart disease, by 2020 [3]. Patients of depression are suffering from decreased productivity, poor psychosocial status, and decreased quality of life and well-being [4].

The Global Burden of Disease (GBD) 2017 study indicated that mental and behavioral disorders accounted for 22.6% of all years lived with disability (YLDs) [1]. The major category was depressive disorders, with major depressive disorder (MDD) causing 63 million YLDs, and dysthymia causing 11 million YLDs; together accounting for 9.6% of all YLDs [1]. In modern society where fierce competition often occurs, depression has become a social problem that cannot be ignored. Research conducted in young subjects indicated that an increasing prevalence of adolescent or teenage depression may deteriorate this situation even further in the near future. According to a recent study, the cumulative incidence of pubertal depression has increased from 5% to 20% [5,6]. Related studies have shown that the eating habits formed at this stage can also affect teenagers’ emotions and affect their mental health in further years [7].

In the *Practice Guideline for the Patients with MDD* released by American Psychiatric Association (APA), pharmacotherapy, psychotherapy, or combined therapies are recommended. However, medication and/or psychotherapy have limitations. Besides the financial burden, side effects of antidepressant medications also cause severe problems. Common side effects of antidepressant medicines include nausea, increased appetite and weight gain, sexual problems such as erectile dysfunction and decreased orgasm, fatigue and drowsiness, and insomnia, to name a few. Moreover, emerging data also indicate that antidepressants may promote suicide [8]. As such, people are now thinking of using alternative methods for MDD prevention and treatment. One of the most attractive ideas is the consumption of a suitable diet as an alternative. Furthermore, a well-designed diet may help to stop obsessing ones’ bad days, therefore lowering the risks of falling in illness or delaying disease progress.

Balanced nutrition plays an important role in our model of thinking and our behavior, as the intake of foods affects our cognition, memory capacity, and emotions. Besides a balanced diet, isolated nutrients are another element when adopting nutritional strategies to fight MDD. Neurotransmitters are the major subjects, where activated substances in different areas of the brain actively participate in the creation of nerve impulses, thereby regulating our mental abilities and emotions. The interactions between different foods and these neurotransmitters cause different emotions [9]. Researches showed that the foods we eat might affect the chemical composition of our brain, thus changing our mood. For example, ginseng extract G115 and some probiotics, *bifidobacterium adolescentis* NK98 and *Lactobacillus reuteri* NK33, have been shown to attenuate depressional behavior by increasing brain-derived neurotrophic factor (BDNF) contents [10,11]. Vitamin D and its metabolite calcitriol could protect our brain from depletion of dopamine and serotonin, thus contributing to brain health [12,13]. MDD is also accompanied with oxidative stress, therefore antioxidants might contribute to protecting us from MDD [14]. Research into the possible role of dietary factors in depressive symptoms is a common problem in public health [4]. In addition, a project named Multi-country cOllaborative project on the role of Diet, Food-related behavior, and Obesity in the prevention of Depression (MooDFOOD), involving 13 organizations in eight European countries, uses a unique integrative approach to explore the prevention of depression through nutritional strategies. This approach combines expertise in nutrition, consumer behavior, psychiatry, and preventive health psychology. The project has been granted nearly nine million Euros to investigate food intake, nutritional status/intake, food-related behavior, and causality between obesity and depression over a five-year period. The relevant results have been used to develop multinational randomized controlled trials. MooDFOOD will be the first multinational project to implement a feasible and effective nutrition strategy to prevent depression, which will help improve the diets of all European Union (EU) citizens in a sustainable manner, and prevent depression [15]. It can be seen that the world has been appreciating the importance of the relationships between depression and diets or nutrition in recent years.

In brief, MDD poses heavy health and economic burdens, thus it must be treated as a global public health priority. Although there is significant research covering topics in eating and moods, the present narrative review will take a glimpse into the relationships between diet patterns, certain food and nutrients including dietary antioxidants, and depression so as to provide a potential preventive and therapeutic approach for the adjuvant treatment of depression.

## 2. Definition and Some Hypothetical Mechanism of Depression

MDD is a common and serious medical illness that affects how we feel against daily requirements or stressful situations, making us think and act negatively [16]. Though MDD appears to be treatable these days, medical treatment does not apply to every patient, and the side effects are troublesome. Therefore, how to effectively prevent MDD is a crucial question.

Depression could be described as an abnormal negative mood, which can strike anyone and at any time, and in most cases, we can recover spontaneously. Depression can affect anyone, even a person who appears to live in relatively ideal circumstances, whilst females are more likely than males to experience depression [17].

However, when the complicated neurotransmitter network achieves pathological abnormality, patients are encouraged to seek medical suggestions, whilst the first signs usually come into appearance during the late teens to mid-twenties. Adolescence is not only a critical period of depression prevention, but also related to current and future disease conditions, and increases the risk of youth suicide [9]. Etiological hypotheses of depression show that this is a disease of multiple origins, with several factors playing roles. The first is about biochemistry, where differences in certain chemicals in the brain may contribute to symptoms of depression [18]; whilst the second is genetics. In a population of 758 identical twins and 306 dizygous females, the bivariate structural equation model estimated 20% of depression to be caused by shared genes [19]. A third is personality; where people with low self-esteem who are easily overwhelmed by stress, or who are generally pessimistic, appear to be more likely to experience depression. Environmental factors or medical conditions (e.g., continuous exposure to violence, neglect, abuse or poverty, or thyroid or brain dysfunction) may also make some people more vulnerable to depression [20,21,22,23,24]. 

Social and psychological stress from daily life, abnormal endocrine secretion, and consequences of drug consumption are all involved with the occurrence of depression, where it has always been a big challenge to reveal the underlying neurobiological, cellular, and molecular mechanisms. Stress from daily life may influence our brain through several interconnected routes, thus leading to depression. Stress also plays a role in precipitating depression, through regulating pathways in the sympathetic and parasympathetic nervous systems and inflammatory responses that could also be induced by the stress [25,26]. Inflammatory responses play an important role in the pathophysiology of numerous mental illnesses including depression. In animal studies, toll-like receptors (TLR) are representative innate immune receptors that mediate with inflammatory responses. TLR2- and TLR4-deficient mice failed to show social defeat stress-induced behaviors, indicating that the innate immune system plays a key role in MDD evolvement and pointing out the importance of anti-inflammatory treatment on mitigating depression [27]. In fact, previously, increased levels of typical inflammatory cytokines and chemokines, such as interleukin (IL)-6 and/or C-reactive protein are frequently observed in serum and/or plasma of depressed patients [28,29,30]. To be precise, during depression, the brain’s immune cell microglial cells are activated by switching on TLR2 and TLR4, inducing afterward inflammatory responses and leading to depressive symptoms [27]. In postmortem studies using human brain, the highest numbers of activated microglia were found in those who had been depressed [31,32]. Moreover, the blood–brain barrier (BBB) of depressed patients is weaker, leading to high permeability; which favors more monocytes/macrophages or neutrophils to infiltrate from the blood into brain tissues, and to brain structures including the prefrontal cortex (PFC), hippocampus (HIPP), and nucleus accumbens (NAc) [33,34,35,36]. All these brain regions are highly interconnected, highlighting the complexity of depression treatment. 

Chronic stressors may remodel our brain, leading to lower neurogenesis in indicated brain regions, thus leading to chronic, continuous pathological changes in multiple functional zones including the PFC, HIPP, and nucleus NAc. As mentioned previously, BDNF, an important regulating protein, is reported to not only contribute to depression, but decrease during chronic depression, causing a vicious circle and deteriorating depression over time [37]. In fact, lowered serum BDNF was found in MDD patients in several randomized controlled trials or meta-analyses, and is suggested to be an indicator of the efficacy of antidepressant medications [38,39,40,41]. Moreover, the decrease of BDNF also induced chronic inflammation and other damaging effects in the indicated brain regions [42,43,44,45]. Stressors and cytokines both increase hypothalamic (and extrahypothalamic) corticotropin-releasing hormone (CRH) release, activating bombesin-like peptides. Subsequently, with a gamma-aminobutyric acid (GABA) receptor as the mediator, CRH may influence 5-hydroxytryptamine (5-HT) secretion as well. Abnormal 5-HT production is also involved with the pathological processes of depression, or indirectly impairs neuroplastic processes. During these processes, it is worth noting that inflammation may also be induced, and then subsequently influence the cytokine-depression cycle through numerous pathways. One of them is the NF-κB pathway, from where MAPK, JAK/STAT, JNK, or ERK may be activated; and depression-related cytokines such as tumor necrosis factor (TNF)-α, IL-1β, and IL-6 production may increase [46,47,48]. Serotonin signaling, CRH, arginine vasopressin, bombesin, leptin, and GABA are the main keystone transmitters regulating this process. These would again favor impaired neurofunction, aggravating MDD [49,50,51].

The Father of medicine, Hippocrates, once said: “Let your food be your medicine, and your medicine be your food”. The improving effects of diets on depression may be concluded as the following three hypotheses: (1) Diets with anti-inflammatory properties may relieve the inflammatory cytokine secretion, attenuating the inflammation in the brain, thus contributing to depression relieve; (2) Diets with antioxidative properties may decrease the oxidative stress in specific brain zone, thus alleviating depression symptom; and (3) Diets with enhancing ability of BDNF may directly improve patient conditions through up-regulating of BDNF. In brief, depression originates from multiple origins, from social and psychological stress, or from immunological or neurotransmitter abnormality. Further work is needed both with large sample sizes and by using interaction approaches to further elucidate these processes. In recent years, the dysfunction of the central monoamine neurotransmitter system in the physiological and pathological basis of depression has been considered to be the most representative. Dietary nutrition as the primary source of energy for human body functions also has direct or indirect effects on the brain health. The intake of different foods is directly involved in the synthesis and metabolism of related neurotransmitters and has an important influence on human psychology and emotions. Therefore, the relationship between diet and dietary nutrition and depression warrants discussion.

## 3. Diets and Depression

Diets, including dietary patterns or specific foods, and nutritional approaches are closely linked with depression. Dietary patterns refer to combinations of various foods that are combined and consumed in various forms [52]. Moreover, some specific food categories including beverages, (e.g., coffee, tea, carbonated drinks), fresh fruits and vegetables, fish, dairy, and chocolate are found to be related to the cause of depression and pathological processes underpinning depression [53,54,55,56]. We will discuss how dietary patterns and specific foods interact with depression in the following parts.

### 3.1. Dietary Patterns and Depression

Dietary patterns are usually alternatively defined as the number, type, proportion, or combination of different foods and drinks in the diet, and the frequency with which they are habitually consumed [57]. In general, researchers have two ways to determine the dietary patterns. First is the priori approach, which is based on an existing dietary guide or other scientific dietary advice; comparing the individual’s actual diet with the dietary guide or advice to measure compliance. The second is a posteriori approach, which is based on dietary survey data, using statistical methods such as factor analysis and cluster analysis to determine the types of dietary patterns. The three most commonly used dietary patterns in nutrition epidemiology studies are the Mediterranean dietary pattern (MD), the Western dietary pattern (WD), and the Oriental dietary pattern. The relationships between the first two dietary patterns and depression have received great attention [58].

Balanced dietary patterns such as the Mediterranean diet (MD) have been uniquely associated with a lower risk of depression or depressive symptoms in two observational prospective studies and one randomized controlled trial [59,60,61,62]. The last trial, using fish oil as a supplement, obtained a positive result. In this study, the control subjects were inclined to choose “healthier” foods, and a significant reduction of depression was observed (*t* = −2.24, *p* = 0.03). At the same time, mental health scores were improved by MD plus fish oil supplement (*t* = 2.10, *p* = 0.04). The effect of this supplemented diet lasted for 6 months. Sánchez-Villegas et al. later found that supplementing the MD with nuts had a beneficial effect on the depressive risk of diabetes mellitus (DM) 2 patients through the Cox regression model [63]. A project abbreviated as SMILEs (Supporting the Modification of lifestyle In Lowered Emotional States), focusing on a personalized intervention approach for patients with adult depression showed that a modified MD can reduce the depressive symptoms of adult depression in clinical practice [64]. However, it has been noted that as the first randomized control trial, the SMILEs trial seems to fall short: other researchers have pointed out a considerable number of design defects and ill operation during recruitment all lead to an unreliable conclusion [65]. In a cohort study of Australian women, six dietary patterns were studied for cooking vegetables, fruits, the MD pattern, meat and processed meats, dairy products, and high-fat high-glucose diets. It was found that only the MD pattern could reduce the incidence of depression for 3 years [59]. Another study investigating the relationship between the MD pattern with treating cardiovascular disease had a similar effect on the prevention of depression [60]. To the opposite, the WD has been considered as an aggravator for depression. In *The Seguimiento Universidad de Navarra/University of Navarra follow-up* (SUN) cohort project, 493 depression cases out of a total number of 8964 participants were reported. It was reported that a higher risk of depression was associated with consumption of fast food in typical WD. (fifth vs. first quintile: hazard ratio (HR) = 1.36; 95% CI 1.02, 1.81; *p* trend = 0.003) [66]. In fact, saturated fats and refined carbohydrates in a typical WD may induce inflammation and oxidative stress, disturb gut microbiome and gut–brain interaction, cause hippocampus degeneration, and lead to nutrient inadequacies, which are all risk factors of depression [67,68,69]. In fact, a newly conducted meta-analysis about dietary inflammatory index (DII) and depression shed new light on what to eat to fight against depression and why a MD is a better choice than WD during this situation [70]. In this study, four prospective cohorts and two cross-sectional studies enrolling a total of 49,584 subjects have been analyzed. Individuals in the highest DII showed a higher risk of depression than those in the lowest DII category (risk ratio (RR) = 1.23; 95% CI 1.12, 1.35). Interestingly, gender-specific analysis showed that this association was observed statistically significant in women (RR = 1.25; 95% CI 1.09, 1.42) but was not in men (RR = 1.15; 95 % CI 0.83, 1.59). The results suggested that a higher DII score is independently associated with an increased risk of depression, particularly in women [70]. 

Obviously, aside from these two dietary patterns, other dietary patterns have also been reported to successively regulate mood. Adherence to a diet rich in vegetables, fruits, and typical Japanese foods including mushrooms, seaweeds, soybean products, and green tea is associated with a lower probability of having depressive symptoms [71]. A study using reduced rank regression to explore the relationships between a 9 year dietary pattern and depressive symptoms, found a typical Tuscan diet rich in vegetables, olive oil, grains, fruit, fish and moderate in wine and red and processed meat might be protective against depressive symptoms [72]. Depressive symptoms were closely related to adolescent dietary patterns [73]. Molendijk et al. collected data from 24 independent cohorts with a total of 1,959,217 participants, and confirmed that adherence to a high-quality diet, regardless of types (i.e., healthy/prudent or Mediterranean) was associated with a lower risk of depressive symptoms over time (odds ratios ranged from 0.64 to 0.78). A relatively low dietary inflammatory index was also associated with a somewhat lower incidence of depressive symptoms (odds ratio = 0.81), although not in a dose-response fashion. Similar associations were found for the consumption of fish and vegetables (odds ratios 0.86 and 0.82, respectively), but not for other high-quality food groups (e.g., fruits) [74]. Adherence to the Traditional Indian Confinement diet by the intake of herbs and beans, and adherence to the Eat-Out diet and Soup–Vegetables–Fruits diet by the intake of fruits, vegetables, and fish have been shown to reduce postpartum depression and postpartum anxiety, respectively [75]. 

There is a growing body of health epidemiological evidence supporting that a dietary pattern which has a higher intake of fruits, vegetables, olive oil, nuts, fish, and whole grain; and a lesser intake of meat, meat products, commercial bakery, trans fat, and sugary dessert/drinks may reduce the risk of depression. Among these dietary patterns, whether differences in the intake of some micro or macronutrients can make a difference in their associations with a lower risk of depression requires exploration [76]. As we discussed above, bad food patterns may spoil our intestinal flora, thus impairing our neuro-endocrine function. A fecal microbiota transplant has been applied in different disorders such as Clostridium difficile infection, irritable bowel syndrome, inflammatory bowel diseases, insulin resistance, multiple sclerosis, and idiopathic thrombocytopenic purpura [77]. The application of fecal microbiota transplants may also show promise as a treatment for depression. 

### 3.2. Specific Foods and Depression

It has been a belief that foods could influence health and well-being. Forthwith, people began to show great interest in how certain foods affected mood and brain function, and medical culinary textbooks described the relationships between them. For example, eggs, peacock, beef, pomegranates, and apples were considered to be erotic stimulants; quince, dates, and elderberries were used as mood enhancers, and lettuce and chicory as tranquilizers [52]. Below we aim to discuss about the associations among fish, fresh fruit and vegetables, and sugar-sweetened drinks and depression.

#### 3.2.1. Fish Consumption and Depression

Many have reported that infrequent fish consumption may lead to depressive symptoms decades ago [78,79]. In the past 3 years, several studies verified this correlation. Grosso et al. examined 31 studies, including 255,076 individuals and over 20,000 cases of depression. Analysis of 21 datasets investigating the relationship between fish consumption and depression displayed significantly reduced risk (RR = 0.78, 95% CI: 0.69, 0.89) [54]. Li’s meta-analysis [55] also indicated that high fish consumption could reduce the risk of depression. For depressive symptoms during pregnancy, similar negative correlations were also found between fish intake and depressive symptoms [56]. Interestingly, gender seems to play an essential role in the association of fish consumption and depression. In Finnish adults, women who consume fish more than once a week compared with those who do not showed less depressive symptoms (27.0 percent versus 34.2 percent; χ^2^ = 9.05, *df* = 1, *p* < 0.01) [80]. In another study in Northern Finland, risk of developing depression increased up to 2.6-fold (95% CI 1.4–5.1) among rare fish eaters (less than once a month) when compared with regular eaters [81]; In Australian young adults (26–36 years old), women who ate fish ≥2 times/week at baseline had a 25% lower risk of depression during follow-up than those who ate fish <2 times/week (adjusted relative risk = 0.75, 95% confidence interval: 0.57, 0.99) [82]. However, in the above studies, none had reported that depression was associated with fish consumption in males [80,81,82].

#### 3.2.2. Fresh Fruit and/or Vegetable Consumption and Depression

Fresh fruit and vegetables are rich in nutrients, including antioxidants. It is reported that fruits or vegetables may modify brain serotonergic status and have a positive effect on mood, as with other carbohydrate-rich foods [83]. In a large-scale national survey of Canadians, including 8353 participants aged over 18 from 2002/2003 to 2010/2011; participants were asked to fill out a questionnaire about daily fruit and vegetable consumption, physical activity, smoking behavior, depression, and psychological distress symptoms. The results showed negative correlations between fruit and vegetable consumption per cycle and depression in the next cycle (β = −0.03, 95% CI −0.05 to −0.01), and psychological distress (β = −0.03, 95% CI −0.05 to −0.02) [84]. Liu’s team also found significant negative correlations between fresh fruit and depressive symptoms, but significant positive correlations between ready-to-eat food (fast foods) and depression symptoms [85]. Wurtman and Wurtman suggested that carbohydrate could relieve depression [86]. In fact, in 2019, there was an up-to-date umbrella review of observational studies, and meta-analyzed correlation between fruit and vegetable consumption and overall health outcomes. In this study, possible evidence was found that fruit or vegetable intake, respectively, is associated with decreased risk of depression [87]. Another meta-analysis validated this conclusion: ten studies involving 227,852 participants for fruit intake, and eight studies involving 218,699 participants for vegetable were included. An inverse association of fruit (0.83 (0.77, 0.91; *p* = 0.006)) and vegetable (0.88 (0.79, 0.96; *p* = 0.007)) intake with risk of depression was observed [88]. Though studies adopted various classification standards based on complicated demography, it is recommended to consume fruits or vegetables as frequently as possible based on the existing data [83,84,85,86,87,88]. 

#### 3.2.3. Sugar-Sweetened Drink Consumption and Depression

It was reported that sugar intake was associated with depression because it altered endorphin levels and oxidative stress [89]. Modern food industries are exploiting our food preferences with sugary drink; however, evidence has shown that there is the association between sugar-sweetened soft drink consumption and depression. A cross-sectional survey was conducted with 3667 adults in Tianjin, China, where dietary intake was assessed using a valid self-administered food frequency questionnaire [90]. Depressive symptoms were assessed using the *Zung*’s Self-Rating Depression Scale (SDS). The results showed that the odds ratios (95% CI) of having elevated depressive symptoms by increasing levels of soft drink consumption were 1.00, 1.43 (1.01, 2.01), and 2.00 (1.15, 3.37) after adjustments for potential confounding factors [90]. Prospective analyses regarding the associations of sugar intake from sweet food/beverages and recurrent mood disorders showed that sugar intake from sweet food/beverages was positively associated with recurrent depression after 5 years (highest vs. lowest tertile odds ratio: 1.81; 95% CI: 1.23, 2.66) [91]. In the youngsters, a survey conducted among 8226 Chinese students consuming soft drinks ≥7 times/week had significantly higher (mean difference; 95% CI) depression scores compared with those barely consuming soft drinks (two-item Generalized Anxiety Disorder: 0.15; 0.07, 0.23 (mean difference; 95 % CI); two-item Patient Health Questionnaire: 0.27; 0.19, 0.35) [92]. A meta-analysis of 10 observational studies summarized that the consumption of sugar-sweetened beverages might be associated with an increased risk of depression, while the threshold was the equivalent of about 2 cups/day of cola, above which the depression risk might be increased obviously [93]. However, in another study conducted among 15,416 Spanish university graduates, although participants in the highest quartile of added sugars consumption showed a significant increment in the risk of depression (HR = 1.35; 95% CI 1.09, 1.67, *p* = 0.034), no significant association between sugar-sweetened beverage consumption and depression risk was found [94]. Based on the mixed results, further study is encouraged about the association between sugared drinks and depression, and the result may depend on the carbohydrate contents.

### 3.3. Food Addiction and Depression: Extend Your Menu to Enrich Your Brain Health

The Yale Food Addiction Scale (YFAS) is a tool to determine the relationships between food addiction and mental health symptoms. A meta-analysis covering 51 studies using the YFAS indicated that the mean prevalence of food addiction diagnosis was 16.2%, the mean number of food addiction symptoms in populations seeking treatment for weight loss was 3.01 (range 2.65–3.37), and this was higher in groups with disordered eating (mean 5.2, range 3.6–6.7). There were significant positive correlations between depression and food addiction (mean *r* = 0.459 (0.358–0.550)) [95]. In another study about food addiction and depression, a questionnaire called *Night Eating Diagnostic Questionnaire* was employed to discuss the relationships among depression, sleep quality, “food addiction”, and body mass index. Students (*n* = 254) and community members (*n* = 468) were administered the Night Eating Questionnaire, NEDQ, Pittsburgh Sleep Quality Index, *Zung*’s Self-report Depression Scale (SDS), and the YFAS. It was indicated that higher NEDQ score was associated with higher SDS risk and YFAS score, indirectly indicating that the risk of depression and the potential of being food-addicted have a common alternating pattern [96].

These findings suggest that depression might be inextricably linked to many single foods. However, in nutritional epidemiology, two different situations need to be distinguished. Changing depression states can sometimes be associated to a single food, but in real life, people cannot eat only one kind of food in a meal, and often consume many kinds of food at the same time. Therefore, there may be interactions between foods, such as synergy or inhibition. The effect of a single food or nutrient may also be eliminated by other foods or nutrients. In this way, the relationship between a single food and depression may be a superficial phenomenon, rather than a real one. Focusing on a single food will be one-sided, and a single assessment cannot take into account any interaction between different foods. Within this context, the method based on the dietary patterns should be a more scientific and accurate approach. The dietary patterns go beyond foods and cover the impact of the overall model, while capturing the various potential interactions between different foods. Therefore, it is more meaningful to study overall dietary patterns compared with single foods. As inflammation plays large roles in the pathological progress of depression, it should be addressed that some specific foods with anti-inflammatory or antihyperlipidemic properties are promising in depression prevention and treatment. For example, specific fiber (rice bran) is reported for its antihyperlipidemic function in high-fat diet-induced brain inflammation and dysfunction, while short chain fatty acids (SCFAs) have been shown to attenuate neuroinflammation in various models [97,98]. The supplementation of omega-3 polyunsaturated fatty acids (PUFA), particularly eicosapentaenoic acid, can reduce the incidence of depression [99,100,101]. However, intraventricular infusions of propionic acid, a kind of SCFA, induced oxidation and inflammation, which may induce a pathological status [102]. The efficacy of these foods needs further investigation.

## 4. Nutrients and Depression: Focusing on Dietary Antioxidants

It is well known that depression is associated with defective antioxidant defenses and increased levels of serum superoxide dismutase (SOD) and serum malondialdehyde (MDA), and decreased levels of plasma ascorbic acid and Vitamin E are also found in patients with MDD [103,104]. In fact, it is reported that antioxidants intake is lower in adults with depression [105]. Therefore, the focus of research into the possible role of nutrition in depressive symptoms has involved nutritional ingredients, especially antioxidative nutrients such as n-3 PUFA, folic acid, vitamin B12, vitamin D, polyphenols, and so on in recent years.

A meta-analysis of 14 studies comparing the levels of PUFA between patients with depression and controls, found that the levels of EPA, DHA, and total n-3 PUFA were significantly lower in depressed patients than in control individuals [100]. There was no significant change in Arachidonic acid or total n-6 PUFA. The results confirmed that n-3 PUFAs played an important role in the pathogenesis of depression [101]. As we discussed above, there was a significant negative correlation between the incidence of depression and per-capita fish consumption [78,79,80,81,82]. Although the data can provide the evidence for the negative relationship between depression and n-3 PUFA, the causal relationship between them cannot be determined. Due to PUFAs being closely related with depression, many clinical trials have been conducted to explore the effect of n-3 PUFA on depression. For example, except for consuming their normal antidepressant medications, patients were provided with n-3 PUFAs (EPA 740 mg and DHA 400 mg) daily or placebo daily for 16 weeks [106]. The results showed that the depressive symptoms of patients who were taking n-3 PUFAs had been significantly improved, according to Brief Psychiatric Rating Scale (BPRS) total score (treatment-by-time interaction, *p* = 0.0184) [106]. In addition, other clinical trials have shown that omega-3 PUFA is more effective than placebo [107,108,109] or as effective as conventional antidepressant medication fluoxetine [110] in treating patients with major depression. Besides its antioxidative properties, the antidepressant effect of n-3 PUFAs may be also related to the adjusting capacity of serotonin system [111], proinflammatory cytokines [112,113], and BDNF [114,115]. Although the mechanism underlying the associations between n-3 PUFAs levels and depressive symptoms is not fully understood, a review by Su et al. suggested that four factors might be involved; neurotransmitters, inflammation, oxidation, and neuroplasticity [116]. For example, n-3 PUFA and their counterparts, n-6 PUFA might be related to depression via inflammation pathways. The n-6 PUFA can be precursors for the pro-inflammatory series of eicosanoids, whereas n-3-derived metabolites are precursors for the anti-inflammatory series of eicosanoids [115]. 

Folic acid is one of the vitamin B complexes and belongs to the same carbon unit as vitamin B12 and homocysteine; whilst high homocysteine levels are usually caused by lack of folic acid, vitamin B6, or B12. Folic acid and vitamin B12 are involved in the process of single-carbon metabolism, which is directly related to the production of monoamine neurotransmitters and other important methylation reactions in the brain. Studies indicated that low levels of folic acid in the body were associated with higher rates of depression [117]. Later studies showed that not only folic acid, but low levels of vitamin B12 in plasma or high homocysteine levels were also associated with an increased risk of depression [118,119,120]. The reason may be that the low levels of folic acid and vitamin B12 lead to high levels of homocysteine, which is an amino acid containing sulphur that is proatherogenic, prothrombotic, and cytotoxic through its tendency to increase oxidative stress, inducing DNA strand breakage and apoptosis [121]. However, studies investigating the effects of folic acid on depression have produced inconsistent results. In a study of women’s health and aging [122], serum levels of homocysteine and folic acid and the prevalence of folate deficiency were not associated with depression status, but metabolically significant vitamin B12 deficiency was associated with a two-fold risk of severe depression. Due to the close relationships between folic acid/vitamin B12 and depression, folic acid supplements have long been used for the treatment of depression, and diets rich in folic acid and vitamin B12 could significantly reduce the risk of depression [123,124,125].

Studies revealed that the occurrence of depression was associated with the abnormal increase of Ca^2+^ in neurons [126]. Epidemiological evidences showed that vitamin D deficiency was associated with 8–14% increase in depression [127,128,129] and a 50% increase in suicide rate [130]. Similarly, vitamin D deficiency was found to be linked with depression in the young [131]. Mood symptoms in depression were improved after treatment with vitamin D [132,133]. Vitamin D could improve depression, mainly by regulating Ca^2+^ concentration and serotonin synthesis. Vitamin D could increase expression of the plasma membrane Ca^2+^-ATPase (PMCA) and Na^+^/Ca^2+^ exchanger 1 (NCX1) that extrude Ca^2+^, and the calbindin D-9k, calbindin D-28k, and parvalbumen that buffer Ca^2+^ [134,135]. In addition, vitamin D could also induce the expression of serotonin hydroxylase 2 in the serotonin synthesis gene, inhibiting the expression of tryptophan hydroxylase 1 at the same time. Therefore, vitamin D could prevent depression by maintaining normal serotonin levels [136]. 

Plant polyphenols and their antidepressant properties have been recently highlighted. Tea polyphenols, one of the most widely spread antioxidants, were reported for their antidepression effects in a human trial [137]. In this study, a total of 537 Japanese men and women aged 20–68 years were recruited. Higher green tea consumption was associated with a lower prevalence of depressive symptoms. Compared with participants consuming ≤1 cup/d, those consuming ≥4 cups green tea/d had a 51% significantly lower prevalence odds of having depressive symptoms (*p* for trend = 0.01) [137]. A meta-analysis concluded that a borderline nonsignificant association between tea consumption and risk of depression was found (RR 0.70, 95% CI 0.48, 1.01) [56]. In another study carried out in midlife and older women, 82,643 women without a previous history of depression at baseline were recruited. After a 10 year follow up, a total of 10,752 incident depression cases were reported. Inverse associations between flavonoid intakes and depression risk were observed (*p*-trend = 0.0004). Higher intakes of all flavonoid subclasses except for flavan-3-ols were associated with significantly lower depression risk; flavones and proanthocyanidins showed the strongest associations (HR for both: 0.83; 95% CI: 0.77, 0.90) [138]. In 2018, an Italian study reported that dietary intake of phenolic acid (OR = 0.64, 95% CI: 0.44, 0.93), flavanones (OR = 0.54, 95% CI: 0.32, 0.91), and anthocyanins (OR = 0.61, 95% CI: 0.42, 0.89) showed significant inverse associations with depressive symptoms in 1572 adult Southern Italian dwellers in a dose-response manner [139]. In a cross-sectional study published in 2019, higher dietary phytochemical intake was reported to be associated with lower prevalence of depressive symptoms in a total of 488 female participants (OR 0.22; 95% CI 0.12, 0.38) [140]. Antioxidants may improve depressive symptoms, even in a one-time administration. In another study, children and young adults were asked to consume a flavonoid-rich blueberry drink, and mood was assessed using the Positive and Negative Affect Schedule before and 2 h after consumption of the drinks. As a result, acute blueberry intervention increased positive effects [141]. As for specific kinds of antioxidants, higher intake of dietary isoflavones was independently related to a lower prevalence of depressive symptoms during pregnancy in a total of 1745 Japanese pregnant women (95% CI 0.46–0.86, *p* = 0.002) [142]. Animal studies are often used to study mechanisms. Apigenin flavonoids are widespread in plants, they are reported to have sedative, neuroprotective, and antidepressant effects through the antagonism of glutamatergic N-methyl-d-aspartate (NMDA) and GABA pathways [143]. Icariin could affect the limbic–hypothalamic–pituitary–adrenal axis interaction with serotonergic; thereby, regulating the central corticotropin-releasing factor system and exerting its antidepressant effect [144]. The total flavonoids of *Scutellaria baicalensis* could inhibit blood viscosity and raise the viscosity of viscous plasma in depressive rats. The changes of the functions of free radicals and antioxidant enzyme defense systems were associated with the onset of depression, and there were more lipid peroxidation products in patients with severe depression [145]. A recent experiment revealed that resveratrol regulated the hippocampal Wnt/β-catenin pathway, as well as decreasing depressive behavior in rats [146]. After intervention with procyanidins in a depressed rat model, the expression of phosphorylated cAMP response element binding protein (p-CREB) and BDNF increased significantly in the HIPP and prefrontal cortex. Thus, procyanidins could improve depression and anxiety like behavior in rats by enhancing the cAMP–CREB–BDNF signal transduction pathway. In addition, studies have shown that lemon essential oil, pectin, caffeine, red ginseng saponins, D-serine, and so on are related to improving depression. Lemon essential oil has antidepressant-like effects through inducing a modulation effect on both the serotonergic and dopaminergic systems in the brain, especially in the striatum and hippocampus [147]. Pectin can improve depression, which may be due to the influence of IL-6 concentration and JAK–STAT signaling pathway in hippocampus of mice [148]. Caffeinated coffee reduces depression-like behavior, observed 24 h after Lipopolysaccharide (LPS) administration through an anti-inflammatory pathway [149]. Rg3, one of the most popular saponins from red ginseng, exerted beneficial effects in systemic inflammation-induced depressive-like behavior in mice. These protective effects were partially achieved through the inhibition of neuroinflammatory disturbance and the regulation of TRP–KYN metabolism both in the brain and in the periphery [150]. D-serine plays an antidepressant role through rapidly activated AMPA–MTOR signaling pathway [151]. However, we have to admit that though animals may present depression-like behaviors, animal studies are not paralleled with human trials. The efficacy of a potential antidepressant should be validated using human species.

To summarize, natural antidepressants have become a trend in the development of new antidepressant drugs. Due to their safety and low toxicity, phytochemicals could be a good source of natural antidepressants in the future, and they are now widely used in the development of antidepressant drugs. 

## 5. Conclusions

As people in modern society are burdened with tremendous pressure, the incidence of mental illness is increasing. Studies have shown that diet and nutrition play a significant role in the prevention and clinical treatment of depression, implying that the concepts of diet and nutrition could be incorporated into future depression intervention programs. Diet and nutrition can be used as a part of a comprehensive strategy for the prevention of depressive problems. Moreover, patients with depression who are not suitable for drug therapy or psychotherapy can use diet and nutrition adjustments as an alternative treatment. Therefore, future research should focus more on understanding the efficacy and dose responses of foods and dietary patterns upon depression and explore the effects of different dietary patterns for different types of depression patients. Meanwhile, there is a crucial need to deliver better education for the public and clinicians about the role of diets and nutrients in sustaining mental health. Further attention should be paid to establish a diet therapy for the prevention, complementary treatment, and maintenance of mental health by focusing on not only foods, but also the combination with exercise and favorable lifestyle factors in the future [151,152].
